# Supplementary material for: Synthetic soil crusts against green-desert transitions: a spatial model
Source: R Soc Open Sci. 2020 Aug 26;7(8):200161. doi: 10.1098/rsos.200161 (PMC7481726; doi:10.1098/rsos.200161)
Supplement: Supplementary Information [file rsos200161supp1.pdf]

Synthetic soil crusts against green-desert transitions: a spatial model

Supplementary Information

Blai Vidiella[1,2], Josep Sardanyés[3,4] , Ricard Solé[1,2,5,*]

1. ICREA-Complex Systems Lab, Department of Experimental and Health Sciences, Universitat Pompeu Fabra, Dr. Aiguader 88, 08003 Barcelona, Spain
2. Institut de Biologia Evolutiva (CSIC-Universitat Pompeu Fabra), Pg. Marítim de la Barceloneta 37, 08003 Barcelona, Spain
3. Centre de Recerca Matemàtica. Campus de Bellaterra, Edifici C 08193 Cerdanyola del Vallès, Barcelona, Spain
4. Barcelona Graduate School of Mathematics (BGSMath). Campus de Bellaterra, Edifici C 08193 Cerdanyola del Vallès, Barcelona, Spain
5. Santa Fe Institute, 1399 Hyde Park Road, Santa Fe, NM 87501, USA

∗ corresponding author

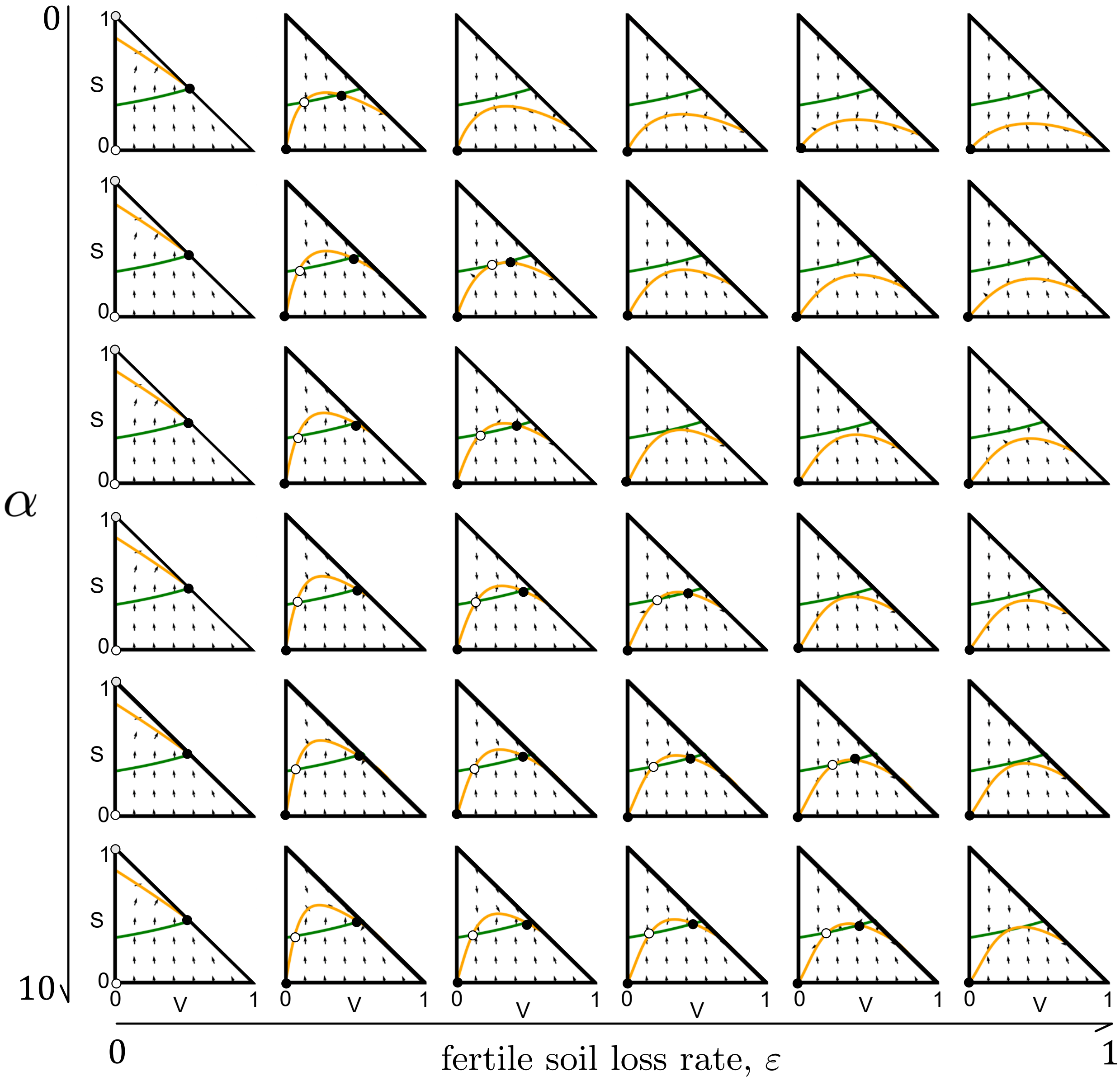

FIG. S1: Shape of the nullclines computed numerically from Eqs. (3.5) (green line) and (3.6) (orange line) of the main manuscript at increasing ε and α values. The directions of the flows are indicated with small arrows. Here the intersections of the nullclines are fixed points (black: stable; white: unstable). Depending on parameter values different equilibria are found: P_1^* at the origin; P_2^* at the vertical axis of the phase space and both P_3^* (interior saddle point) and P_4^* (interior stable node). The dynamics and basins of attraction can be seen in figure S2. The other parameters are fixed at: $r = 0$, $f = 0.9$, $\delta = 0.1$, $b = 0.6$, $c = 0.3$, $m = 0.15$, and $\beta = 0$

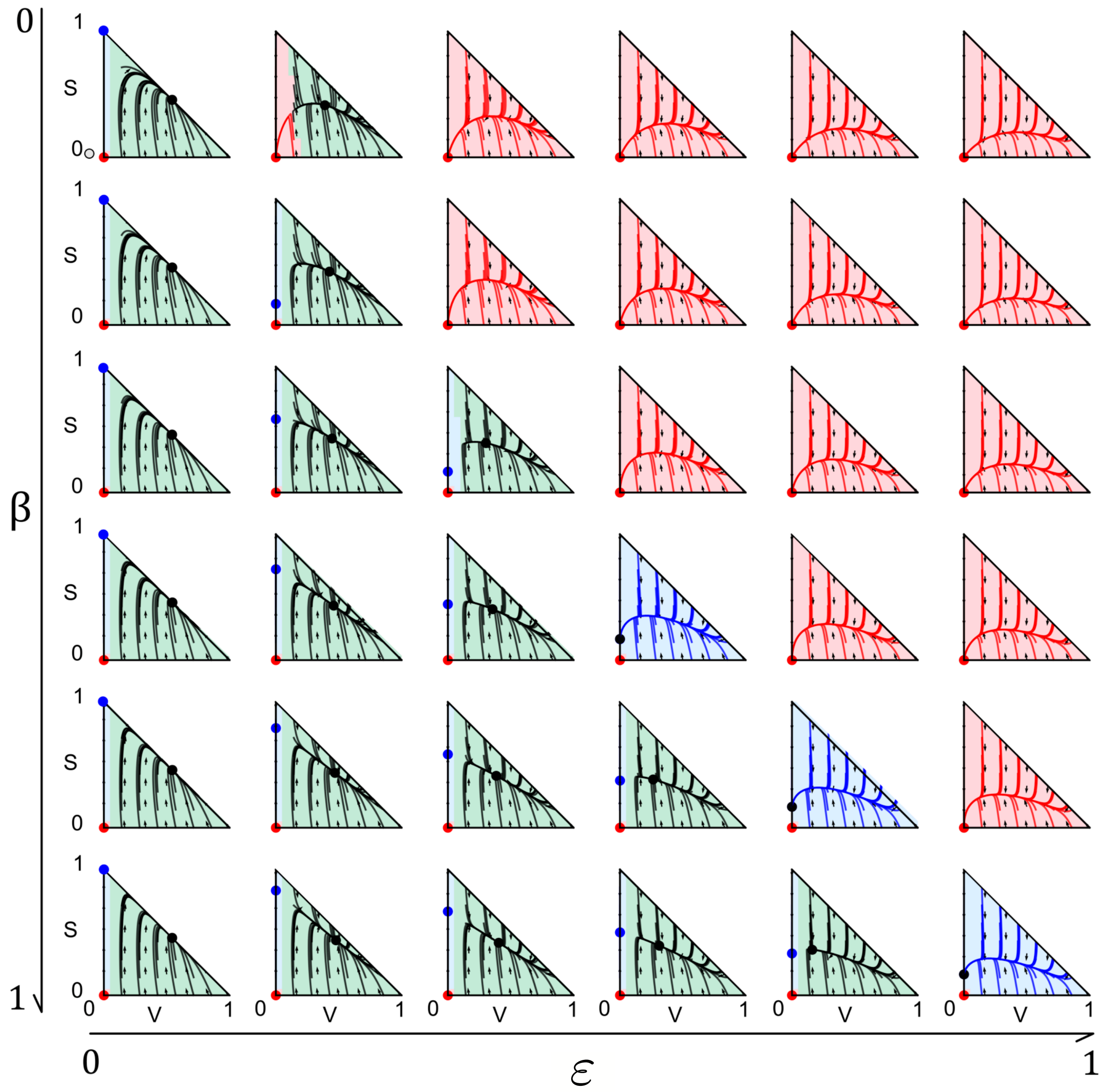

FIG. S2: Dynamics within the phase space (V, S) shown in Fig. S1, here with some orbits and the basins of attraction. The directions of the flows are indicated with small arrows. Basins of attraction of each fixed point are displayed with different colours: coexistence of vegetation with fertile soil (green); full desert state (red); coexistence between fertile and desert soil without vegetation (blue). Note the orbits are also shown with the colours of the basins they move in. The parameters are the same as in Fig.S1.

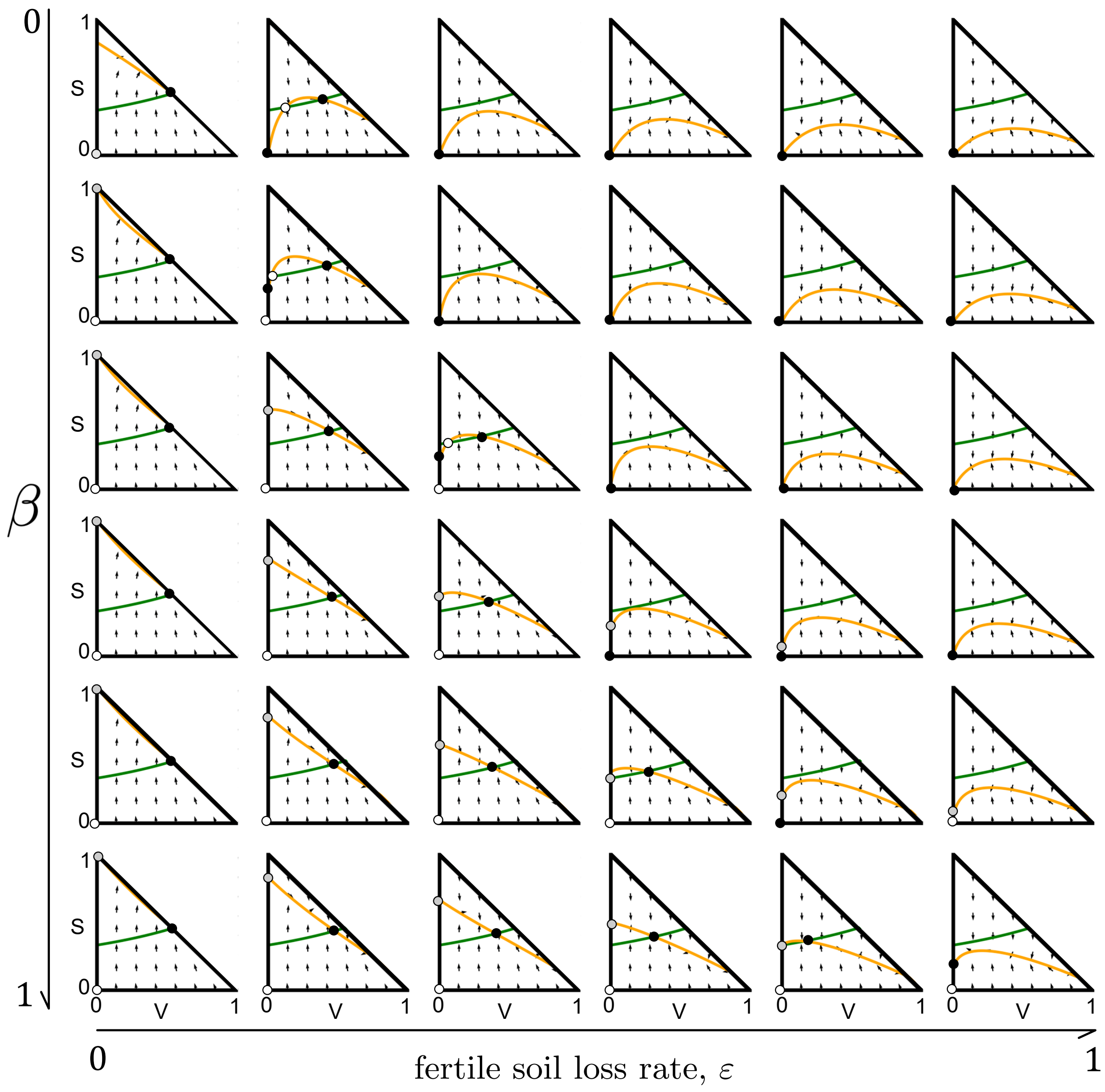

FIG. S3: Same as in Fig. S1 now increasing ε and β, with $\alpha = 0$ and keeping all other parameters as in Fig. S1.

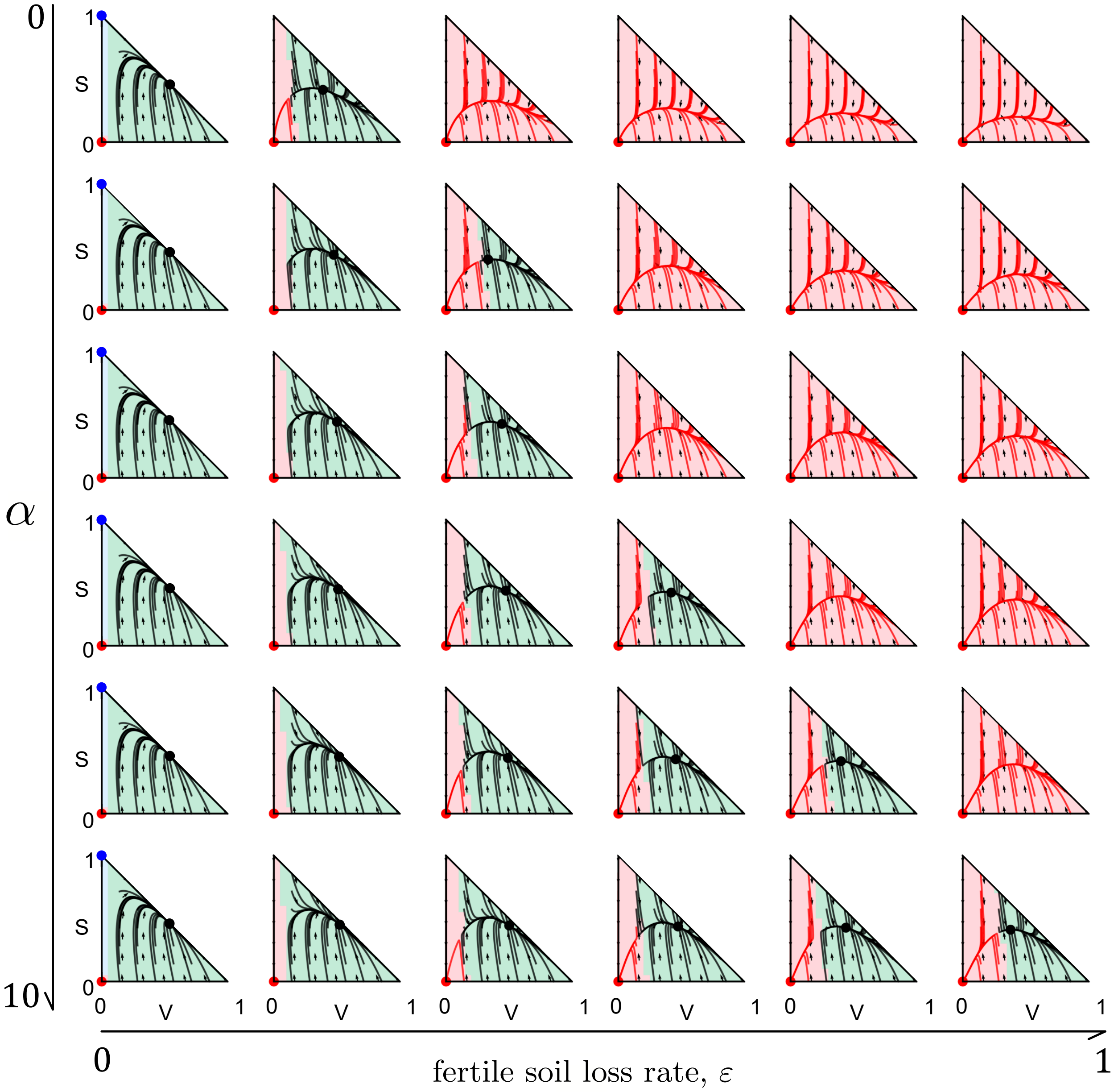

FIG. S4: Same as in Fig. S2 for the cases shown in Fig.S3, now increasing ε and β with $\alpha = 0$, and keeping all parameters as in Fig.S1.

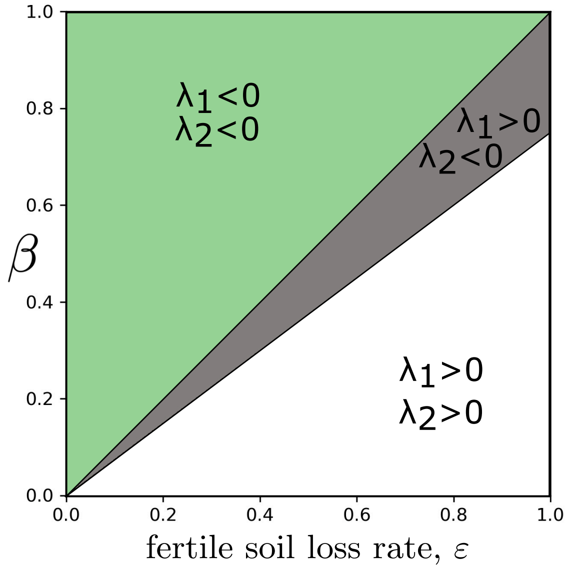

FIG. S5: Local stability of the fixed point P_2^* (with eigenvalues $\lambda_1 = d - \beta$ and $\lambda_2 = b(1 - d/\beta) - m$) in the parameter space (ϵ, β). This equilibrium is stable if $\lambda_1, 2 < 0$ (green region). The grey region indicates a saddle point, being locally stable in the S-axis and locally unstable in the V-axis. Finally, the white region displays the parameter values in which this equilibrium is a repeller, with $\lambda_{1,2} > 0$.

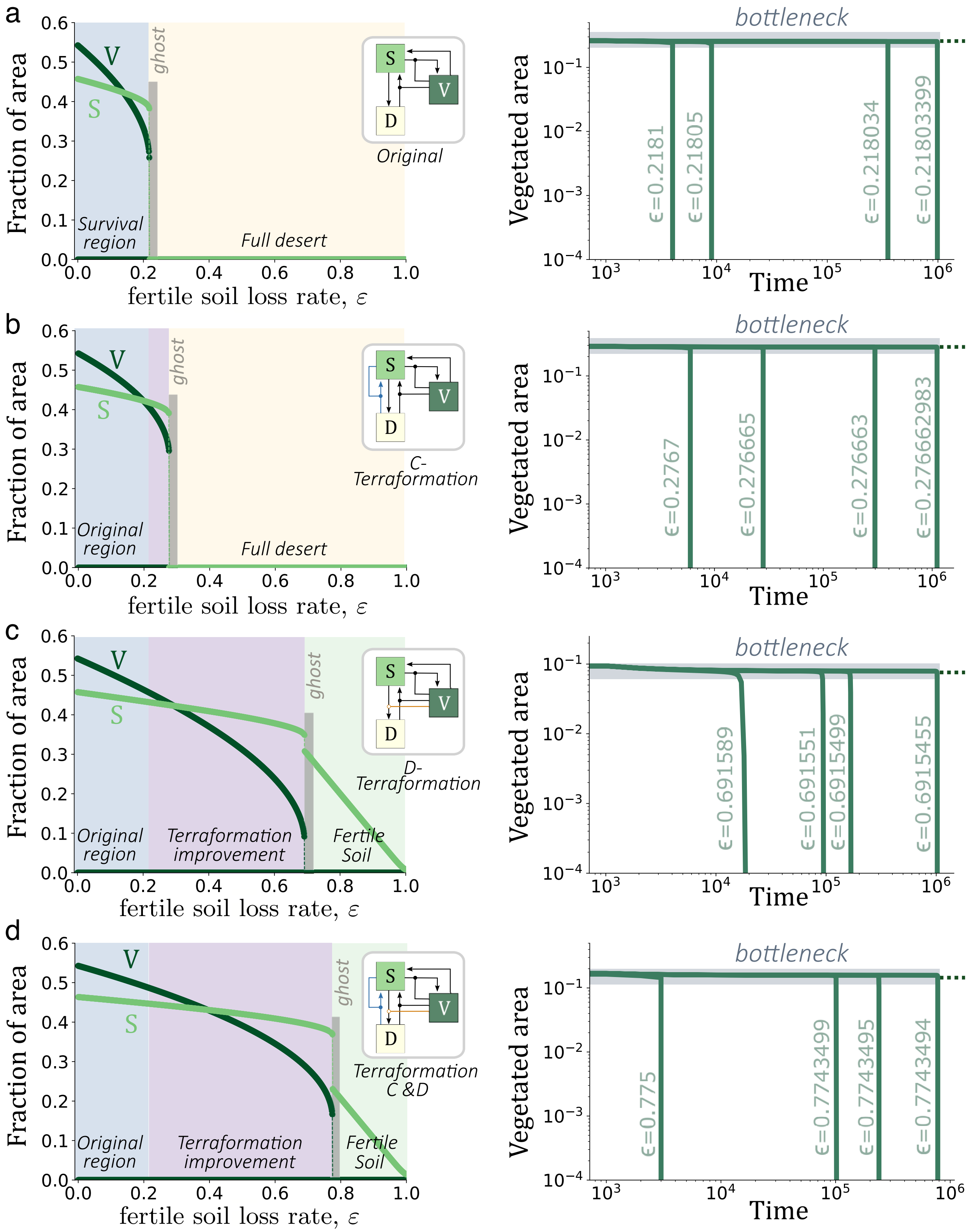

FIG. S6: (a-d) Bifurcation diagrams obtained numerically from Eqs. (3.5-3.6, see main manuscript) using ε as bifurcation parameter and $(V(0) = 1, S(0) = 0)$ as initial conditions. On the right column we display time series for the fraction of vegetated area. Each diagram is built for different cases of the terraformation strategies by using different α and β values, except for (a) that has not been terraformed. The displacement of the critical value of ε due to terraformation is indicated with a light-violet area in the bifurcation diagrams.Note that the in all panels the vegetation becomes extinct due to a catastrophic transition at increasing ε. This transition is given by a saddle-node bifurcation between the interior equilibria P_2^* and P_3^*. After the transition two possible states can be achieved: desert dominance ((a) and (b) with $\alpha = 1$ and $\beta = 1$) or coexistence between fertile soil and desert without vegetation ((c) and (d), with $\beta = 1$). All the time series show the time evolution of the vegetated area: just after the bifurcation they experience a very long transient before extinction: a *dynamical ghost* or delayed transition. Dark dashed lines indicate the equilibrium value for vegetation (V^*) just before the transition.The parameters are the same as in Fig. S1.

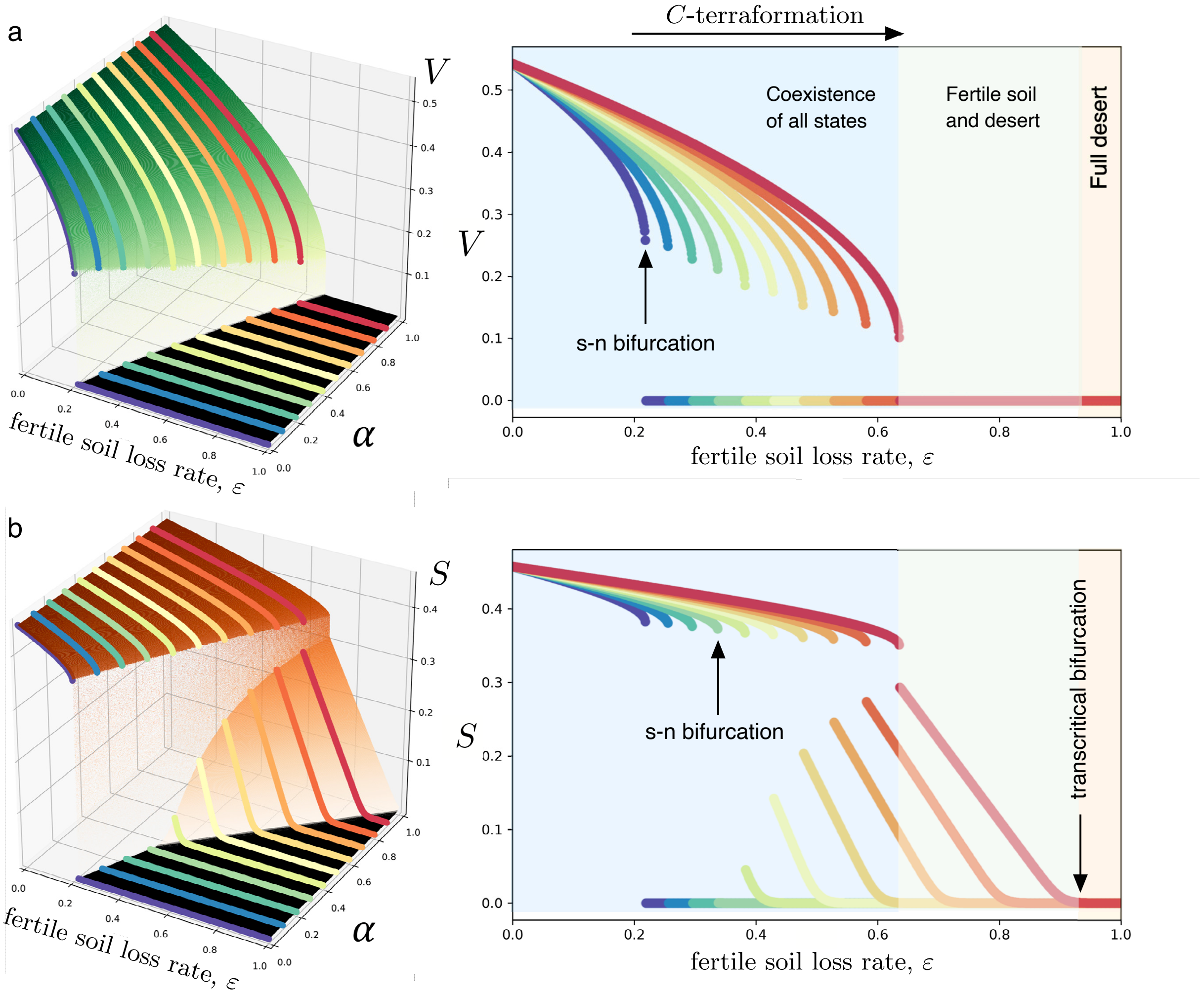

FIG. S7: Equilibria of vegetation (a) and fertile soil (b) displayed in the parameter space (α, ε) keeping $\beta = 0$. We also display the change in the equilibrium states for the consecutive cuts shown on the surface (indicated with different colours fixing the value of α and ε). The bifurcation diagrams on the right display the equilibria at increasing the degradation rate of the fertile soil, ε, for different values of α (using the same colour patterning than the 3D plots). We have labeled the different bifurcations that the system suffers at increasing ε: saddle-node (s-n) and transcritical bifurcation. The transparent blue area shows the values for which vegetation coexists with the fertile soil. The transparent green area means no vegetation and persistence of fertile and desert soil. The transparent orange area corresponds to the full desert state. The other parameters are fixed as in Fig. S1.

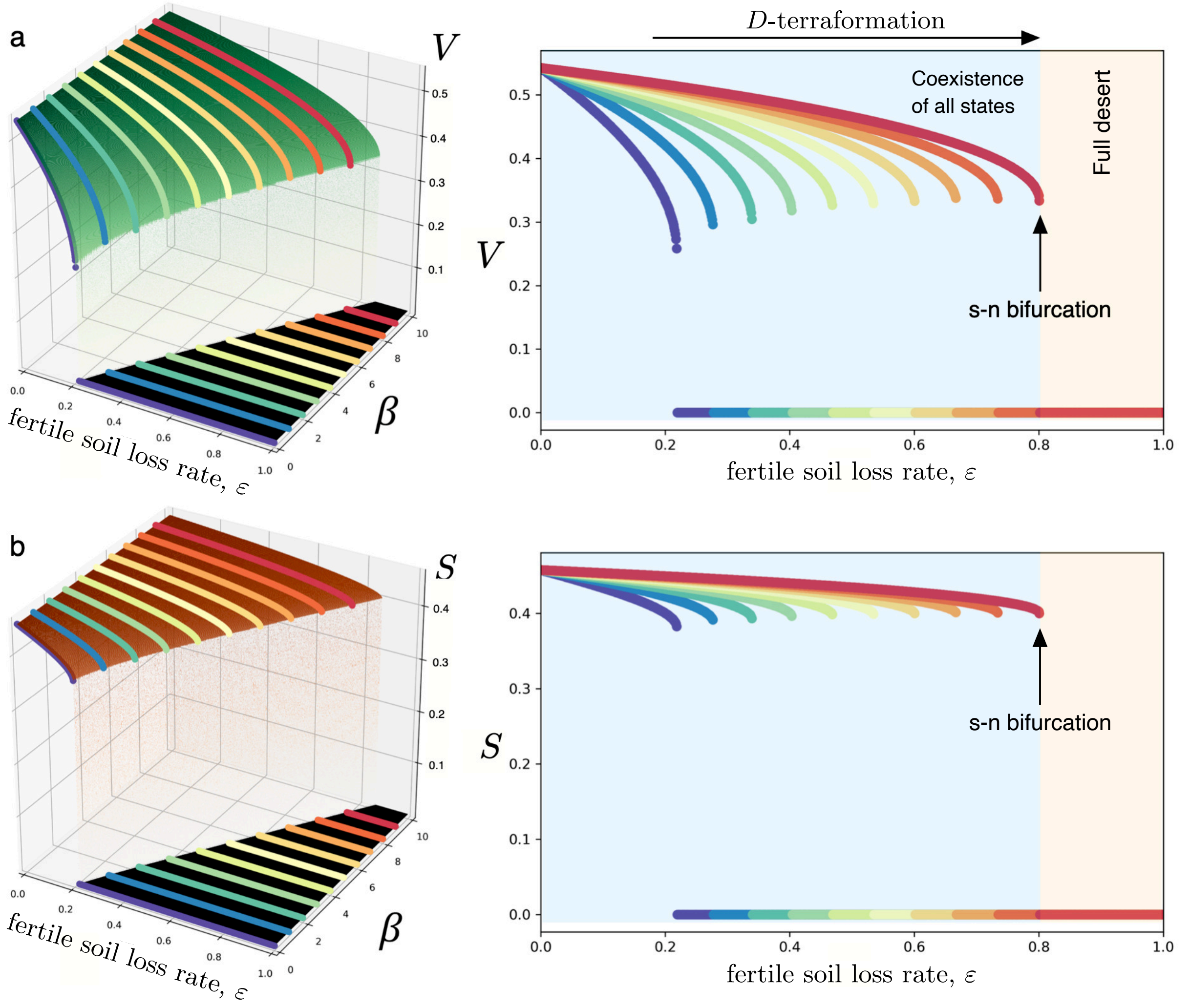

FIG. S8: Same as in Fig.S7 now shown in the parameter space (β, ε) with $\alpha = 0$. The other parameters are fixed as in Fig. S1.

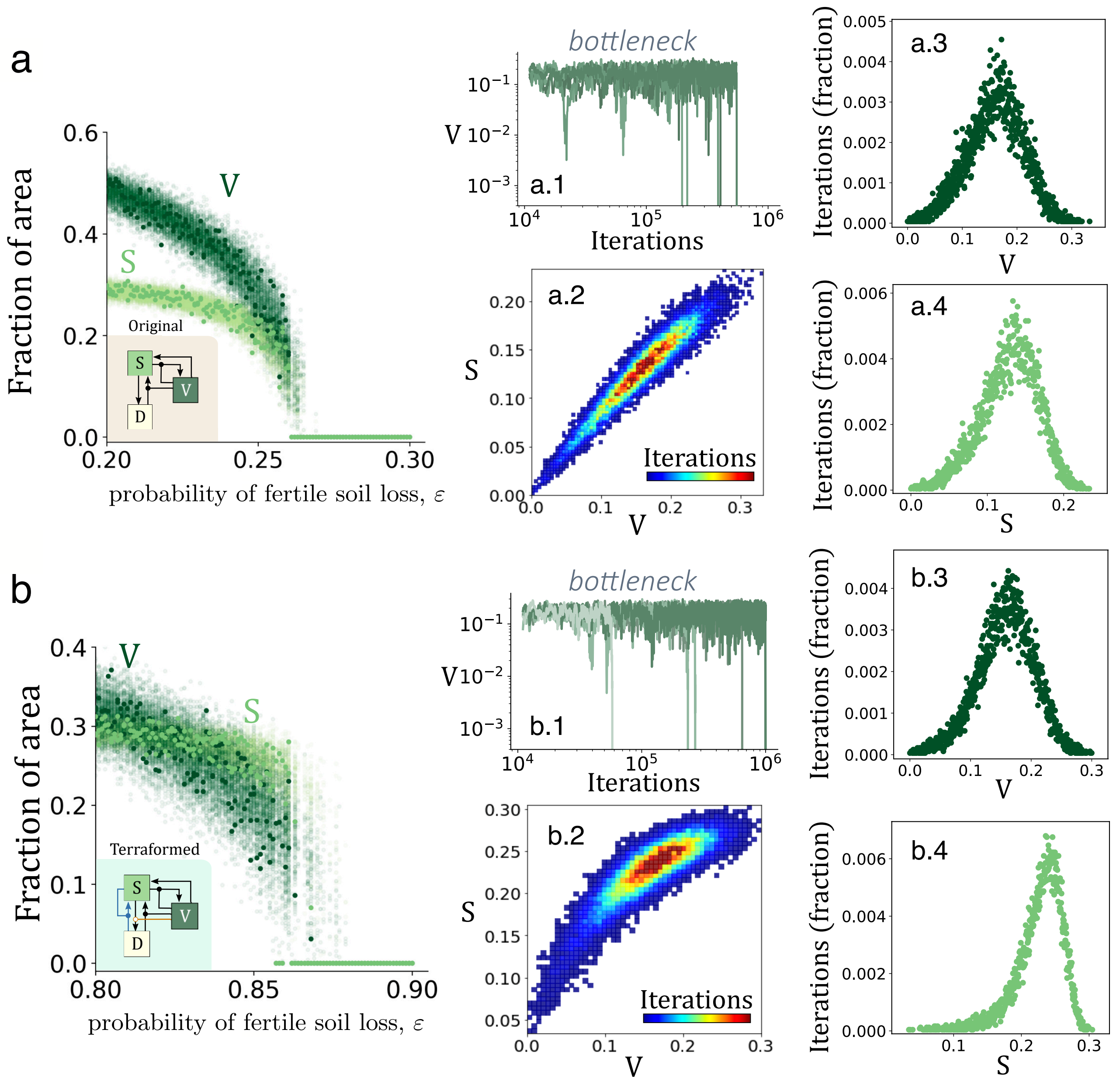

FIG. S9: Dynamical properties near the transition found in the spatial system (50 × 50 sites lattice). (a) Fraction of vegetated area (V) and of fertile soil (S) at increasing ε for the non-terraformed system (the critical value is $\varepsilon_c \approx 0.26$) computed following the same procedure than in Fig. 4 in the main manuscript. Here fluctuations are larger due to the the smaller lattice size. After the extinction, the vegetation suffers an extremely long delay due to a ghost (see the time series for the mean field model in Fig. S6). (a.1.) Five realisations with extinction of vegetation close to the transition. Note that the bottleneck, despite the level of noise, is here also clearly visible. Panel (a.2) displays the region in the phase space (V, S) where the system spends more time to achieve the full desert state, indicating where the ghost is found. Panels (a.3) and (a.4) display the fraction of iterates accumulated at a given density. Here the peak of the distribution corresponds to the value of the variables found at the bottleneck regions of the ghost. (b) Same as in (a) for the terraformed system ($\alpha = \beta = 1$, here with $\varepsilon_c \approx 0.86$). All other parameters are fixed as in Fig. S1.
